# Focally administered succinate improves cerebral metabolism in traumatic brain injury patients with mitochondrial dysfunction

**DOI:** 10.1177/0271678X211042112

**Published:** 2021-09-08

**Authors:** Abdelhakim Khellaf, Nuria Marco Garcia, Tamara Tajsic, Aftab Alam, Matthew G Stovell, Monica J Killen, Duncan J Howe, Mathew R Guilfoyle, Ibrahim Jalloh, Ivan Timofeev, Michael P Murphy, T Adrian Carpenter, David K Menon, Ari Ercole, Peter J Hutchinson, Keri LH Carpenter, Eric P Thelin, Adel Helmy

**Affiliations:** 1 Division of Neurosurgery, Department of Clinical Neurosciences, University of Cambridge, Cambridge, UK; 2 Division of Neurosurgery, St. Michael’s Hospital, University of Toronto, Toronto, Canada; 3 Department of Neurosurgery, The Walton Centre, Liverpool, UK; 4 Department of Chemistry, University of Cambridge, Cambridge, UK; 5 Medical Research Council Mitochondrial Biology Unit, University of Cambridge, Cambridge, UK; 6 Wolfson Brain Imaging Centre, Department of Clinical Neurosciences, University of Cambridge, Cambridge, UK; 7 Division of Anaesthesia, Department of Medicine, University of Cambridge, Cambridge, UK; 8 Department of Clinical Neuroscience, Karolinska Institutet, Stockholm, Sweden; 9 Department of Neurology, Karolinska University Hospital, Stockholm, Sweden

**Keywords:** Cerebral metabolism, microdialysis, mitochondrial dysfunction, succinate, traumatic brain injury (Human)

## Abstract

Following traumatic brain injury (TBI), raised cerebral lactate/pyruvate ratio (LPR) reflects impaired energy metabolism. Raised LPR correlates with poor outcome and mortality following TBI. We prospectively recruited patients with TBI requiring neurocritical care and multimodal monitoring, and utilised a tiered management protocol targeting LPR. We identified patients with persistent raised LPR despite adequate cerebral glucose and oxygen provision, which we clinically classified as cerebral ‘mitochondrial dysfunction’ (MD). In patients with TBI and MD, we administered disodium 2,3-^13^C_2_ succinate (12 mmol/L) by retrodialysis into the monitored region of the brain. We recovered ^13^C-labelled metabolites by microdialysis and utilised nuclear magnetic resonance spectroscopy (NMR) for identification and quantification.

Of 33 patients with complete monitoring, 73% had MD at some point during monitoring. In 5 patients with multimodality-defined MD, succinate administration resulted in reduced LPR(−12%) and raised brain glucose(+17%). NMR of microdialysates demonstrated that the exogenous ^13^C-labelled succinate was metabolised intracellularly via the tricarboxylic acid cycle. By targeting LPR using a tiered clinical algorithm incorporating intracranial pressure, brain tissue oxygenation and microdialysis parameters, we identified MD in TBI patients requiring neurointensive care. In these, focal succinate administration improved energy metabolism, evidenced by reduction in LPR. Succinate merits further investigation for TBI therapy.

## Introduction

Following traumatic brain injury (TBI), a number of pathological mechanisms can inflict damage to central nervous system (CNS) cells.^1^ Within the clinical domain, the emphasis of therapeutic interventions in severe TBI has been on correcting physiological derangements, such as hypotension, hypoxia and raised intracranial pressure (ICP), i.e. conditions that compromise the ability of CNS cells to generate energy.^2^ While there have been several iterations of the Brain Trauma Foundation guidelines for management of severe TBI (most recently 2017),^3^ the primary focus remains on treating raised ICP. The recent BOOST-II (Brain Oxygen Optimization in Severe Traumatic Brain Injury Phase-II) trial incorporated brain tissue oxygenation (PbtO_2_) monitoring in a randomised fashion to existing TBI treatment algorithms, and demonstrated that in the group with additional PbtO_2_ monitoring, better cerebral oxygenation was achieved and there was a trend towards a more favourable functional outcome.^4^ This has led to consensus guidelines on TBI management with both ICP and PbtO_2_ monitoring.^5^ This approach has advantages but may still be limited by its focus on a single metabolic derangement (tissue hypoxia) in addition to ICP. Furthermore, even when oxygenation is adequate, ATP generation by mitochondria is central to brain metabolism,^6^ and mitochondria are damaged following both acute (e.g. stroke, TBI) and chronic (e.g. Parkinson’s and Alzheimer’s diseases) CNS pathologies.^7–10^ We have, therefore, set out to identify mitochondrial function in clinical TBI.

**Figure 1.**
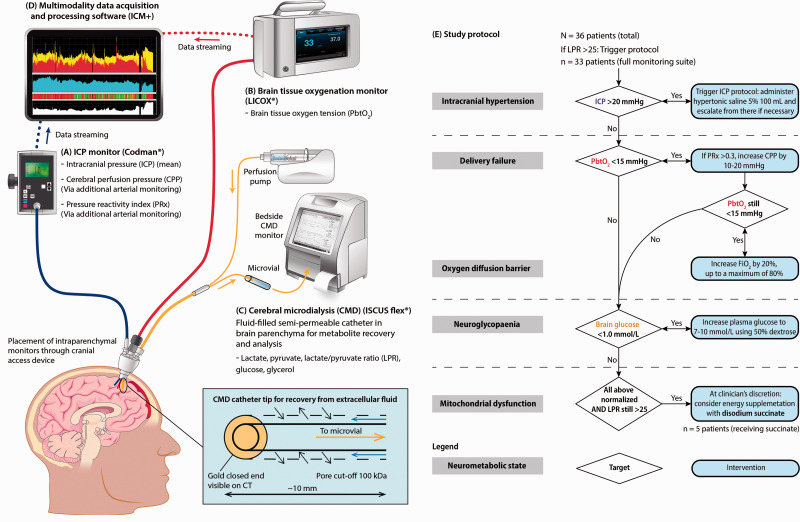
Clinical Monitoring Schema and Protocol. Three intraparenchymal monitors are placed in the sedated, ventilated traumatic brain injury patient, via a cranial access device into the right frontal lobe. (A) Intracranial pressure is measured using a piezoelectric strain gauge (Codman). (B) Brain tissue oxygen is measured using a modified Clark electrode (LICOX). (C) The cerebral microdialysis catheter (M Dialysis AB) consists of a double lumen catheter with a semipermeable membrane (M Dialysis 71, polyarylethersulfone membrane 10 mm length, 100 kDa nominal molecular weight cut-off) at the tip. A microfluidic pump perfuses the catheter with artificial brain extracellular fluid at 0.3 µL/h. The fluid recovered is collected in a microvial and assayed for lactate, pyruvate, glucose and glycerol (bedside ISCUSflex analyser). If the perfusion fluid is supplemented with exogenous molecules (in this study we used disodium 2,3-^13^C_2_ succinate 12 mmol/L), the gradient across the microdialysis catheter is reversed and these molecules can diffuse into the brain: this is termed retrodialysis. At the same time, metabolites arising from the exogenous molecules can diffuse into the microdialysis catheter and be collected in the emerging microdialysate. (D) Signals from intracranial pressure and brain tissue oxygen monitors is streamed in real time to a bedside computer with a multimodality data acquisition and processing software (ICM+) for analysis. Adapted from Khellaf et al., 2019 under Creative Commons License (CC BY),^18^ published by Springer Nature, copyright the Authors. (E) Study protocol for patients with raised lactate/pyruvate ratio. Patients with cerebral LPR>25 were treated in a staged fashion with the interventions within this flowchart. The neurometabolic state was classified in any given hourly time epoch, depending on the abnormalities defined above. CPP: cerebral perfusion pressure; FiO_2_: fraction of inspired oxygen; ICP: intracranial pressure; LPR: lactate/pyruvate ratio; NMS: neurometabolic state; PbtO_2_: brain tissue oxygen tension; PRx: pressure reactivity index.

The cerebral microdialysis parameter lactate/pyruvate ratio (LPR) provides a fundamental biochemical metric of how effectively mitochondria are oxidising NADH,^11^ and LPR above thresholds (>25 and >40) have been correlated with an unfavourable outcome.^12–15^ Derangements in LPR (in the absence of hypoxia or ischaemia) are considered to be the gold standard clinical marker of impaired mitochondrial function,^16^ supported by evidence from large animal models.^6^ Thus, modern multimodal monitoring of TBI incorporates ICP monitoring, allowing calculation of cerebral perfusion pressure (CPP) and pressure reactivity index (PRx),^17^ PbtO_2_ monitoring, together with cerebral microdialysis which allows identification of neuroglycopaenia as well as quantification of LPR (Figure 1).^18^

Studies have shown that there is a failure of ATP production in a subset of TBI patients at a cellular level independent of delivery of suitable substrates (oxygen and glucose).^19^ This condition has been termed “mitochondrial dysfunction” (MD) and it is a phenomenon increasingly recognised in various cerebral pathologies.^10,20^ Different metabolic patterns of LPR, lactate and pyruvate levels have previously been used to define MD.^13^ However, these have relied solely on cerebral microdialysis-recovered metabolites from the brain extracellular space, and not incorporated other metrics such as raised ICP or low PbtO_2_. Ideally, in order to truly discern mitochondrial dysfunction clinically, all other causes of deranged LPR need to be excluded such that mitochondria have all the necessary pre-requisites for oxidative phosphorylation.^19^

Currently, there is a lack of proven therapies for MD. The immunosuppressive drug cyclosporin A (CsA) has been investigated and shown to be tolerated in randomised trials in TBI,^21,22^ and to decrease LPR 4–5 days following TBI.^23^ However, CsA could not be shown to improve clinical outcomes and has substantial side-effects including organ toxicity, particularly renal dysfunction, and increased risk for infections and lymphoma.^24^ An alternative strategy to address MD is the metabolite succinate. Succinate is an intermediate of the tricarboxylic acid (TCA) cycle that interacts directly with complex II (succinate dehydrogenase, catalyses the generation of dihydroflavin adenine dinucleotide (FADH_2_) in the succinate-to-fumarate step of the TCA cycle) of the mitochondrial electron transport chain (ETC)^25^ and can support compromised mitochondria, by bypassing complex I which is more susceptible to inhibition/damage than complexes II, III and IV.^26^ In experimental sepsis (involving energy dysfunction), succinate infusion prevented fall in liver ATP content, and prolonged survival.^27,28^ There is direct evidence of MD in experimental TBI where reduced activities of pyruvate dehydrogenase and complexes I and IV were found in mitochondria in proximity of lesions, relative to contralateral brain.^29^ Furthermore, in an *in vitro* model of MD using glial cultures treated with a complex I inhibitor (rotenone) which raised LPR and decreased oxygen consumption rate, we showed that supplementation with disodium succinate lowered the extracellular LPR, and increased the oxygen consumption rate.^30^ We have also demonstrated that focal succinate administered to TBI patients through the cerebral microdialysis perfusate can improve (decrease) the LPR,^11,31^ with an enhanced brain redox state.^11^ However, in these studies, patients were not specifically selected for suspected MD on the basis of multimodality monitoring.

This study had two aims: firstly, to assess the feasibility and utility of a systematic protocol to identify, characterize and treat derangements of cerebral microdialysis-derived LPR > 25, within a brain multimodality monitoring regimen (Figure 1). Secondly, to target a novel focally administered intervention, succinate, in patients identified as having MD based on cerebral multimodality monitoring parameters. We hypothesised that a tiered protocol of intervention could identify patients with deranged LPR who would benefit from exogenous succinate administration.

## Materials and methods

### Ethics

This study was approved by the Queen Square Research Ethics Committee, London, UK (REC# 17/LO/0587, IRAS# 214040) and registered on clinicaltrials.gov (NCT02993549). Informed written consent was provided by the next-of-kin for each subject. All interventions were discussed with the treating clinician who made the final decision as to implementation of therapies.

### Monitoring and standard treatment regime

We recruited 36 patients with TBI within our centre’s neurocritical care unit between September 2017 and June 2019, of which 33 had the full suite of functioning multimodality monitoring allowing subsequent analyses. Table S1 summarises the clinical and demographic features within this cohort.

All patients were clinically managed using standard neurocritical care unit protocolised ICP control therapy.^32^ In short, these target ICP <20 mmHg using escalating therapy intensity ranging from endotracheal intubation, mechanical ventilation, sedation through neuromuscular blockade, hypertonic saline osmotherapy, maintenance of serum glucose in the concentration range of 5–9 mmol/L, to hypothermia alongside surgical interventions (evacuation of mass lesion, external ventricular drain, decompressive craniectomy). All unconscious TBI patients were managed with multimodal monitoring inserted via a right frontal cranial access device (Technicam Ltd, Newton Abbot, UK) into radiologically normal appearing brain (2014 Cerebral Microdialysis Consensus Guidelines).^16^ Three monitors were utilised: intraparenchymal ICP monitor (Codman, Raynham, MA, USA), Licox PbtO_2_ sensor (GMS, Kiel-Mielkendorf, Germany) and a cerebral microdialysis catheter (M Dialysis 71, M Dialysis AB, Stockholm, Sweden) with 10 mm membrane and 100 kDa pore cut-off. Recordings from ICP/PbtO_2_ monitors and arterial lines (for mean arterial pressure (MAP)) were captured and processed using ICM+ software for Windows (Cambridge Enterprise, University of Cambridge, UK) at the bedside allowing real-time online calculation of PRx, a rolling correlation between MAP and ICP over 10-second ranges.^17,33^ Data was subsequently averaged over one-hour time epochs corresponding to microdialysis monitoring time periods.

Microdialysis catheters were perfused using an M Dialysis 107 pump (M Dialysis AB), at 0.3 µl/minute using CNS Perfusion Fluid (M Dialysis AB). Cerebral microdialysis vials were collected hourly and analysed for brain extracellular glucose, lactate, pyruvate and glycerol using a bedside ISCUSflex analyser (M Dialysis AB). Data points outside the analytical range of the microdialysis analyser were excluded. For ICP and PbtO_2_ monitors, clinical artefacts were curated and removed.

### Clinical study protocol

We conducted a prospective, single-centre, non-randomised interventional study including adult TBI patients in need of multimodal monitoring. We excluded patients with bilateral fixed and dilated pupils, bleeding diathesis, thrombocytopenia (platelets < 100 × 10^3^ per µL), devastating injuries (patient not expected to survive >24 hours), brainstem damage, pregnancy, cerebral microdialysis catheter located in haemorrhagic lesion, and patients younger than 18 years old.

We prospectively employed a tiered management strategy for TBI patients (Figure 1) with microdialysis-derived LPR > 25, based on the following multimodal monitoring parameters: ICP, PbtO_2_, PRx, and brain extracellular glucose. An LPR threshold of 25 was chosen rather than 40 as the higher threshold does not provide additional information in outcome prediction models following TBI.^12,16^ If a patient had LPR > 25 for more than two hours i.e. two consecutive consistent LPR microdialysis samples, the following protocol was employed (Table 1). Each step was attempted for 2 hours before moving on to the next.

**Table 1.** Classification algorithm for neurometabolic states and therapeutic options.

Neurometabolic state	Lactate/pyruvate ratio	Intracranial pressure (mmHg)	Brain tissue oxygen tension (mmHg)	Brain extracellular glucose (mmol/L)	Therapeutic options
Normal LPR	LPR < 25				
Intracranial hypertension	LPR > 25	ICP > 20			ICP control protocol
Delivery failure or oxygen diffusion barrier	LPR > 25	ICP < 20	PbtO_2_<15		Increase CPP by 10–20 mmHgNormobaric hyperoxia
Neuroglycopaenia	LPR > 25	ICP < 20	PbtO_2_>15	Brain Glu < 1	50% dextrose infusion
Mitochondrial dysfunction (MD)	LPR > 25	ICP < 20	PbtO_2_>15	Brain Glu > 1	Succinate by cerebral retrodialysis

Note: Table depicting the classification used for the different neurometabolic states in this study.

Glu: glucose; ICP: intracranial pressure; LPR: lactate/pyruvate ratio; PbtO_2_: brain tissue oxygen tension.

***Intracranial hypertension:*** If ICP >20 mmHg, ICP control measures as per standard clinical protocols (see above).
a) ***Delivery failure:*** If ICP normalised and PbtO_2_ <15 mmHg, and PRx > 0.3, the cerebral perfusion pressure was increased by 10–20 mmHg. b) ***Oxygen diffusion barrier*:** If ICP normalised, PbtO_2_ <15 mmHg, and PRx < 0.3 or CPP had already been raised, the option of normobaric hyperoxia was discussed with the treating clinician.

3. ***Neuroglycopaenia*:** If brain extracellular fluid glucose <1 mmol/L, the plasma glucose levels were increased to a maximum 10 mmol/L using 50% dextrose solution.
4. If all the above steps failed to improve the LPR, and all other parameters were normalised (i.e. ICP <20 mmHg; PbtO_2_ <15 mmHg; PRx < 0.3; brain extracellular glucose >1 mmol/L) we defined this as brain “***mitochondrial dysfunction***” (MD). In these cases, a discussion with the treating clinician was undertaken and only with their permission, disodium 2,3-^13^C_2_ succinate was administered through the microdialysis catheter for 24 hours. For this reason, only a subset of patients with MD were deemed suitable for succinate administration, and in some cases the treating clinician indicated a preference not to administer succinate by microdialysis e.g., during periods of physiological instability or in the perioperative period when monitoring was interrupted. LPR data was collected for the 24-hour epochs before and after succinate administration and compared with the LPR during the 24 hours the patient received succinate.

### Classification of neurometabolic states

Utilising the study protocol (Figure 1), we classified each one-hour monitoring time epoch into an NMS relating to the LPR, namely, normal LPR (<25), intracranial hypertension, delivery failure/oxygen diffusion barrier, neuroglycopaenia and MD. In order to avoid misclassification due to values oscillating around a particular threshold, an NMS was only classified if consistently present for at least two hours. Periods with LPR > 25 not classified within an NMS (pattern persists for only 1 hour) but for which all necessary monitoring data were available were categorised as “inconsistent state”. We excluded from analysis periods where an NMS could not be determined due to lack of necessary monitoring data. Table 1 summarises the characteristics employed.

### Succinate administration

In patients with persistently raised LPR > 25 for greater than 2 hours, despite normalisation of the other multimodal monitoring parameters, i.e. classified as MD, a discussion was had with the treating clinician to determine whether succinate administration by retrodialysis was permissible. The clinical team permitted six administrations of succinate carried out in five patients. Disodium 2,3-^13^C_2_ succinate (isotopic enrichment 99%, chemical purity 99%) from Cambridge Isotope Laboratories, Inc (Tewksbury, MA) was formulated at 12 mmol/L in CNS perfusion fluid by a Good Manufacturing Practice accredited pharmacy (Pharmacy Manufacturing Unit, Ipswich Hospital NHS Trust, Ipswich, UK) and tested for purity, sterility, freedom from endotoxins and absence of pyrogenicity, before release for use in patients. Succinate was administered by retrodialysis of CNS perfusion fluid supplemented with disodium 2,3-^13^C_2_ succinate (12 mmol/L) for 24 h as described by Jalloh et al.^31^ A 2 h exclusion margin was applied to the cerebral microdialysis data to allow for washout/run-in at the start of the 24 h perfusion period and of the post-perfusion period as described previously.^31^ Brain lactate, pyruvate, glucose, LPR, glycerol, PbtO_2_ and ICP were compared between the 24 hours preceding succinate administration (termed pre-perfusion), for the 22-hour period commencing two hours after the start of administration of exogenous disodium 2,3-^13^C_2_ succinate by retrodialysis (termed during perfusion), and for the 22-hour period commencing two hours after the end of succinate administration (termed post-perfusion).

### NMR analysis

After the bedside analysis with the ISCUSflex analyser, brain microdialysate samples were briefly stored at −20°C (for up to 24 hours) and then moved to a -80°C freezer prior to a more in-depth analysis of the biomarkers of brain metabolism using ^13^C-NMR. For the NMR analysis, microdialysate samples of each patient were pooled into 24 h periods during which they received 2,3-^13^C_2_ succinate perfusion, added to deuterium oxide (D_2_O) and internal reference standard (DSS) following the same methodology described previously^31,34^ and transferred into a 3 mm NMR tube (Hilgenberg GmbH, Malsfeld, Germany). ^13^C and water-suppressed ^1^H NMR spectra were acquired on a Bruker Avance III HD 500 MHz spectrometer (Bruker BioSpin GmbH, Karlsruhe, Germany) with a dual ^1^H/^13^C DCH cryoprobe (CP DUL500C/H, Bruker BioSpin GmbH), and TopSpin software (Bruker BioSpin GmbH). ^1^H spectra were acquired using the *zgesgppe* pulse sequence, a 1D experiment that use excitation sculpting with gradients and perfect echo to suppress the water signal.

Acquisition parameters included 32 scans with a 1 second of relaxation delay (d1). ^13^C spectra were acquired using the *zgpg30* pulse sequence, which has a power gated decoupling using a 30ᵒ flip angle on the carbon channel, with 3 seconds of d1 and 4,096 (4 k) scans. For each experiment the receiver gain is set to a constant value. The spectra phase and baseline were automatically and manually corrected using TopSpin 4.0.6 software (Bruker BioSpin GmbH). Identification of metabolite signals was done as previously described.^31,34^

Concentrations [^13^C] of labelled metabolites was determined from the ^13^C NMR spectra and [^12^C] from the ^1^H spectra using the calibration methods described previously.^34^ For single-labelled metabolites, ^13^C has a natural abundance of 1.1%, and ^13^C results for fractional enrichment have been expressed after subtracting this natural background. The probability of having naturally two ^13^C atoms occurring next to each other is 0.01%; therefore, the background for the ^13^C doublet signals has not been subtracted. Fractional enrichment of labelled metabolites can be found as 100 × [^13^C]/([^13^C] + [^12^C]).

### Statistical analysis

Statistical analyses were performed using R statistical software (Version 3.6.0).^35^ For all statistical tests, alpha was set at 0.05 for significance. Patient demographics, as well as their distribution in the different pathological states, are visualised descriptively.

‘Per minute’ data from ICM+ software was averaged over the 1 h periods corresponding to collection points of microdialysis vials as described previously.^12^ After categorising multimodal monitoring data by patient into neurometabolic states, we pooled total durations in minutes, using cerebral microdialysis monitoring time periods, within each NMS by day following injury for all patients to build a contingency table. We then conducted a chi-squared test of independence to evaluate the association between NMS and day since injury using the contingency table and extracted Pearson coefficients to further characterise this association.

After categorisation of neuromonitoring data with NMS, we also extracted frequencies of NMS (% of total) pooled by individual patient over their full respective monitoring period. Shapiro-Wilk tests were employed to test for normality. We performed a one-way analysis of variance (ANOVA) to compare means of groups of individual frequencies of NMS with LPR > 25, i.e. intracranial hypertension, delivery failure/oxygen diffusion barrier, neuroglycopaenia and MD. We performed post-hoc Tukey’s Honest Significant Difference (HSD) tests to further examine differences in mean frequencies between each pair of neurometabolic states.

Changes in ICP, PbtO_2_ and cerebral microdialysis parameters between levels at baseline pre-succinate perfusion, during perfusion, and post-perfusion periods were assessed for statistical significance using a linear mixed-effects model with processed ‘per hour’ data. The lmerTest package, extending from lme4, with fit by restricted maximum likelihood, and ANOVA table of type III with Satterthwaite approximation for degrees of freedom, which provides consistent power,^36,37^ was used.

For the model with LPR as dependent variable, brain glucose, glycerol, PbtO_2_, ICP, MAP, and PRx levels and time in relation to succinate administration (pre/during/post) were used as main random-effect variables. Random intercepts with fixed means for each patient to account for fixed effect by patient and inter-subject variability. Analyte levels were further analysed for differences between the three periods in relation to succinate administration (pre-perfusion/during perfusion/post-perfusion) with post-hoc Tukey Contrast tests for multiple comparisons using the obtained respective mixed effect models.

## Results

### Classification of neurometabolic abnormalities related to raised lactate/pyruvate ratio

We employed the study protocol, detailed in Figure 1, and characterised the specific neurometabolic states (NMS) as defined in Table 1 and these data summarised across all monitoring periods are presented in Table 2. Across all patients, 4/33 had no monitoring periods with LPR > 25, i.e. LPR was consistently <25 throughout all monitoring periods, 4/33 had LPR > 25 in all monitoring periods, and the remainder (25/33) had periods with LPR > 25 and <25. In those categorized neuromonitoring periods with LPR > 25, using the frequency of NMS in individual patient data, there was a statistically significant difference between the frequencies of the different NMS (one-way ANOVA (F(3,128) = 6.47, *p* < 0.001). In particular, we found that MD (i.e. LPR > 25; ICP <20 mmHg at the same time as PbtO_2_ >15 mmHg; PRx < 0.3, brain extracellular glucose >1 mmol/L) was more prevalent on average than both the intracranial hypertension (+15%, *p* = 0.011), and delivery failure/oxygen diffusion barrier (+19%, *p* < 0.001) states. However, the difference between average frequencies of MD and neuroglycopaenia states was not significant (*p* = 0.599).

**Table 2.** Summary of the distribution of neurometabolic states by day following injury.

	Day	Day	Day	Day	Day	Day	Day	Day	Day	Day	Day	Day	Day	Day
	1	2	3	4	5	6	7	8	9	10	11	12	13	14
LPR <25	52.5%	52.2%	63.0%	50.5%	49.1%	38.2%	19.4%	21.7%	15.9%	27.1%	14.2%	14.7%	6.5%	5.3%
Inconsistent state	20.0%	9.3%	7.6%	7.8%	12.2%	12.5%	11.5%	10.2%	10.6%	8.6%	23.8%	11.2%	14.4%	12.8%
Intracranial hypertension	1.0%	4.5%	0.6%	7.2%	1.9%	0.0%	0.8%	3.5%	5.2%	16.4%	13.8%	0.0%	14.8%	19.7%
Delivery failure/	0.0%	2.4%	0.0%	0.5%	3.3%	3.9%	8.6%	0.9%	2.2%	1.8%	0.0%	11.9%	0.0%	0.0%
Oxygen diffusion barrier														
Neuroglycopaenia	1.9%	4.3%	11.1%	14.1%	11.2%	30.8%	40.3%	49.6%	37.2%	27.4%	32.9%	39.5%	35.2%	0.0%
Mitochondrial dysfunction	24.6%	27.2%	17.6%	20.0%	22.3%	14.5%	19.4%	14.1%	28.8%	18.6%	15.3%	22.7%	29.0%	62.2%
Total (h monitoring)	105	372	418	440	366	279	254	209	236	223	148	100	82	20

Note: This table shows aggregate data within each neurometabolic state (percentage of total time) across all monitored patients for each individual day of monitoring. All monitoring periods were corrected to individual time of injury (e.g. day 1: 0–24 hours following individual time of injury). Patients were classified into these groups based on the multimodality monitoring parameters in Figure 1/Table 1. Monitoring periods were only classified if there was a consistent pattern over 2 or more hours, in order to exclude misclassification based on a single spurious value. The total number of hourly samples incorporated is summarised in the last row of the table.

**Figure 2.**
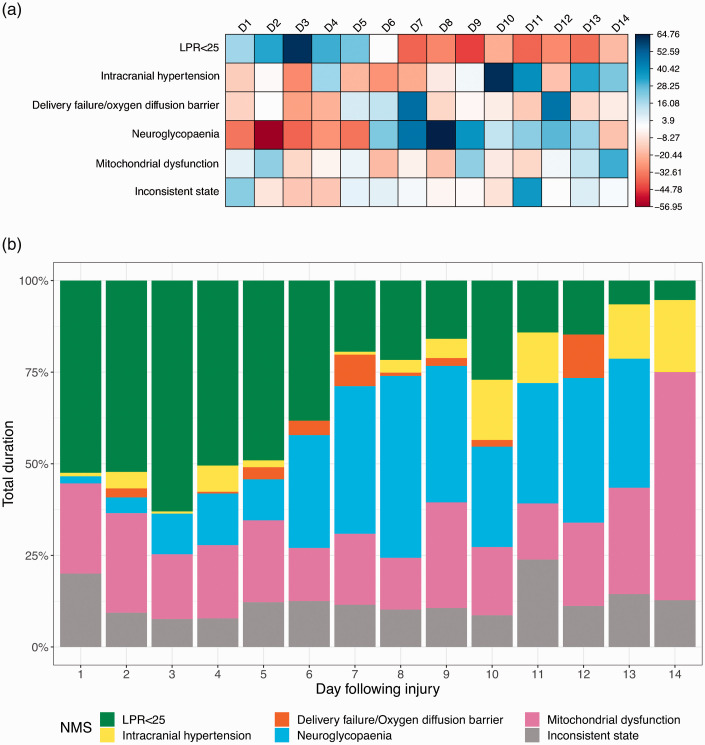
Correlation heat map and histogram of patterns of neurometabolic states following traumatic brain injury. (a) Correlation heat map. The chi-squared (χ^2^) Pearson residuals from the association between neurometabolic state (rows) and day following injury (columns) have been used to generate a correlation matrix for visualisation of the data from Table 2. Positive residuals (blue) indicate a positive association between the corresponding neurometabolic state and day following injury, whereas negative residuals (red) indicate a negative association. Individual cell colour intensity is proportional to the residual correlation coefficient. (b) Histogram. The same data are visualised as a histogram showing the percentage of total time in each neurometabolic state by day following injury. D: day following injury; LPR: lactate/pyruvate ratio; NMS: neurometabolic state.

In total, 24/33 (73%) patients had monitoring periods consistent with MD. We have explored the timing of these abnormalities in relation to the time of injury (Figure 2). There was a statistically significant association between neurometabolic states and day since injury (χ^2^(65) = 56206, *p* < 0.001). Preserved LPR state is most positively associated with the first 5 days following injury, although this may reflect that those patients with normal LPR, and therefore less severe injuries, do not need monitoring beyond this period. However, neuroglycopaenia (NG) was found to be uncommon in the first 5 days and its incidence appeared to peak in days 7–9. Overall, neuroglycopaenia and mitochondrial dysfunction were the commonest NMS in patients with LPR > 25 across all monitoring periods. Following day 12, the data should be interpreted with caution as only one patient was monitored for this long. This reflects the fact that prolonged monitoring is only required in the subset of patients who have persistently deranged neuro-monitoring and may have a more severe pattern of injury.

### Succinate administration

**Figure 3.**
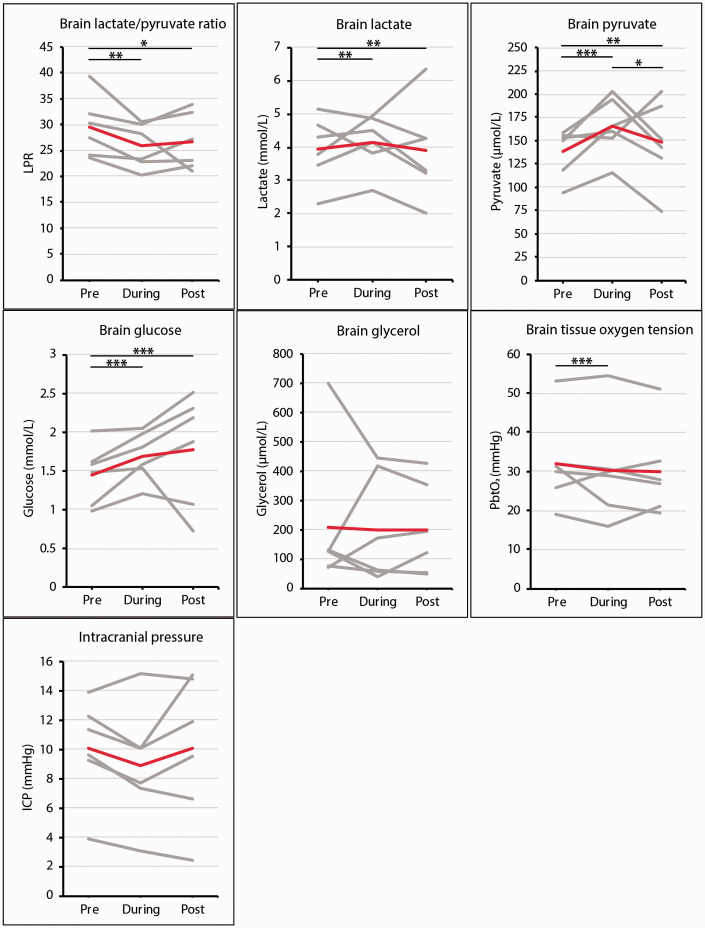
Brain multimodality monitoring data in patients with TBI administered succinate. Mean clinical ISCUSflex cerebral microdialysis analytes, ICP and PbtO_2_ measurements in relation to focal disodium succinate administration. Results are grouped for (I) ‘pre’: 24-hour baseline period (with plain unsupplemented CNS perfusion fluid), (II) ‘during’: 24-hour perfusion period with 2,3-^13^C_2_ succinate disodium salt (12 mmol/L), and (III) ‘post’: 24-hour period post succinate administration (perfusion with plain unsupplemented CNS perfusion fluid). Grey lines represent individual patient data for a specified analyte. A jointed red line represents the mean value for all patients combined for a specified analyte. Bars over lines indicate the significance level by linear mixed effect model analysis of the absolute effect estimates of group comparisons by analyte levels in relation to timing related to succinate infusion, using post-hoc Tukey Contrast tests (*: *p* < 0.05–0.01; **: *p* < 0.01–0.001; ***: *p* < 0.001).

In total, six administrations of succinate were carried out in five patients. The effects of succinate on microdialysis parameters (lactate, pyruvate, LPR, glucose, glycerol) are summarised in Table 3 and Figure 3. In two patients, the mean LPR in the 24 hours preceding succinate administration was <25 as administration of succinate was based on MD identified for a minimum of 2 hours at the end of this period and prior to succinate retrodialysis. There were no adverse events related to focal succinate administration in all involved patients.

**Table 3.** Effect of succinate administration on cerebral microdialysis and multimodality monitoring parameters.

TBI Patient ID	LPR			Lactate (mmol/L)			Pyruvate (µmol/L)			Glucose (mmol/L)			Glycerol (µmol/L)		
	*Pre*	*During*	*Post*	*Pre*	*During*	*Post*	*Pre*	*During*	*Post*	*Pre*	*During*	*Post*	*Pre*	*During*	*Post*
A	24.14	23.39	27.07	2.28	2.69	2.01	94.03	115.73	74.38	1.48	1.54	0.72	130.43	60.90	48.72
B	23.53	20.34	22.15	3.48	4.13	3.24	149.97	203.42	151.05	0.99	1.20	1.07	71.84	173.02	194.33
C1	30.17	28.31	21.06	4.65	3.80	4.26	156.01	152.11	202.20	1.61	1.98	2.31	77.87	57.93	54.14
C2	39.40	30.51	32.29	5.15	4.85	4.25	152.29	160.52	131.69	1.58	1.81	2.19	700.95	445.12	425.61
D	32.12	29.95	33.84	3.77	4.95	6.33	117.91	164.99	186.59	1.05	1.58	1.88	123.84	39.60	123.06
E	27.33	22.85	22.95	4.29	4.50	3.32	157.89	194.94	142.35	2.01	2.05	2.51	128.02	415.34	353.07
Mean	29.45	25.89	26.56	3.94	4.15	3.90	138.02	165.29	148.04	1.45	1.69	1.78	205.49	198.65	199.82
	ICP (mmHg)			MAP (mmHg)			CPP (mmHg)			PRx			PbtO_2_ (mmHg)		
	*Pre*	*During*	*Post*	*Pre*	*During*	*Post*	*Pre*	*During*	*Post*	*Pre*	*During*	*Post*	*Pre*	*During*	*Post*
A	11.35	10.04	15.13	88.30	90.16	97.47	76.95	80.12	82.34	−0.16	−0.26	0.58	31.46	21.49	19.19
B	12.23	10.10	11.93	89.10	92.40	92.36	76.87	82.30	80.43	0.12	−0.02	0.25	19.07	16.06	21.09
C1	9.25	7.68	9.52	75.38	81.92	82.10	66.13	74.25	72.57	−0.27	−0.29	−0.18	25.92	30.03	32.77
C2	9.60	7.33	6.60	85.45	82.71	82.79	75.85	75.38	76.19	0.00	0.02	0.05	31.85	30.71	27.99
D	13.91	15.18	14.81	84.28	98.78	98.53	70.37	83.60	83.72	−0.35	−0.14	0.04	30.08	28.77	26.79
E	3.92	3.03	2.42	75.91	87.86	80.43	71.99	84.83	78.01	−0.13	0.16	0.15	53.11	54.35	51.15
Mean	10.04	8.89	10.07	83.07	88.97	88.95	73.03	80.08	78.88	−0.13	−0.09	0.15	31.92	30.23	29.83

Note: The table summarises each of the microdialysis and multimodality monitoring parameters in the five patients (A-E) who were administered 2,3-^13^C_2_ disodium succinate by retrodialysis. One patient (C) had two distinct administrations of succinate (labelled C1 and C2). *Pre* refers to mean parameters in the 24 hours before administration; *During* refers to the mean parameter for 22 hours during administration (the first two hours of ‘run-in’ within the 24 hours of administration were excluded); *Post* refers to the 22 hours following the administration of succinate (the first two hours were excluded due to wash-out). Patients A and B both showed two (or more) epochs of LPR > 25 prior to recruitment, although had mean LPR < 25 in the 24 hours before succinate administration. The mean values across each of the parameters are summarised below the patient data.

CPP: cerebral perfusion pressure; ICP: intracranial pressure; ID: identification; LPR: lactate/pyruvate ratio; MAP: mean arterial pressure; PbtO_2_: brain tissue oxygen tension; PRx: pressure reactivity index; TBI: traumatic brain injury.

#### 2,3-^13^C_2_ succinate perfusion improves brain lactate/pyruvate ratio by increasing pyruvate

LPR levels during the succinate infusion period were lower than baseline levels (‘pre-succinate’) (mean relative change = −12%; mean absolute change = −3.56; effect estimate = −3.72; *p* = 0.009), a decrease which persisted post-infusion versus baseline (mean relative change = −10%; mean absolute change = −2.89; effect estimate = −3.38; *p* = 0.038). There was no significant difference in effect estimates on LPR levels during versus post succinate infusion (Figure 3). Utilising linear mixed effect model analysis, time in relation to succinate administration (*p* = 0.008), brain glycerol levels (*p* < 0.001) and ICP (*p* = 0.040) were the only factors with a statistically significant effect on LPR throughout the examined period in our patient set with MD. Brain glucose, PbtO_2_, PRx, CPP and MAP levels did not demonstrate a significant statistical effect on LPR. When compared to baseline, reduction in mean LPR levels was mediated by a proportionally higher increase of mean cerebral pyruvate levels (+20%; effect estimate = +20.4; *p* < 0.001) versus lactate levels (+5.3%; effect estimate = −0.312; *p* = 0.009) during infusion.

#### 2,3-^13^C_2_ succinate perfusion increases brain glucose and reduces brain tissue oxygen

Brain glucose was increased during succinate infusion versus baseline (+17%; effect estimate = +0.350; *p* < 0.001), which was sustained post-perfusion versus baseline (+23%; effect estimate = +0.491, *p* < 0.001) (Figure 3). Compared with baseline, there was a small but statistically significant decrease in PbtO_2_ levels during succinate infusion (−5%; effect estimate = −3.33; *p* < 0.001). Mean PbtO_2_ levels remained unchanged pre- versus post-perfusion (−7%; effect estimate = −2.04; *p* = 0.062) (Figure 3). It should be noted that the levels of brain glucose and PbtO_2_ largely remained above clinically established thresholds despite the changes during succinate administration.

#### 2,3-^13^C_2_ succinate is metabolised through the TCA cycle as demonstrated by ^13^C nuclear magnetic resonance

**Figure 4.**
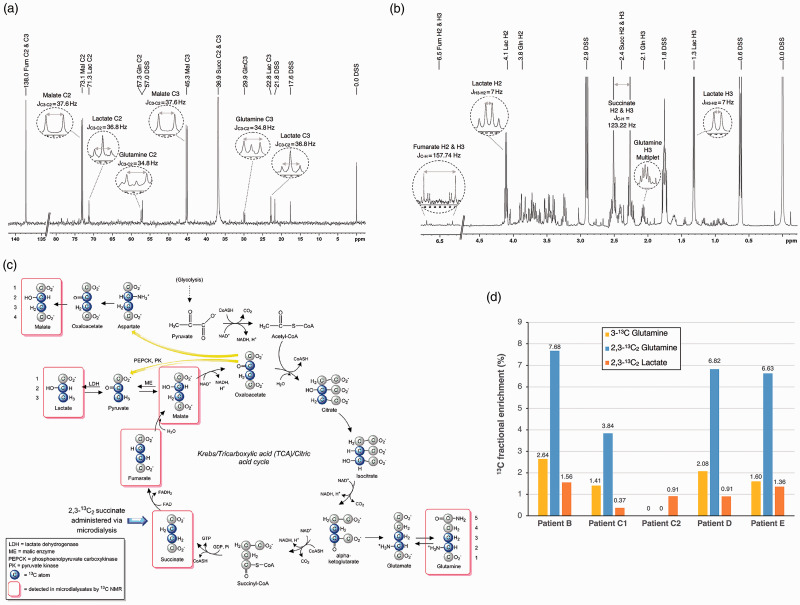
NMR spectra from cerebral microdialysis samples. (a) *Indicative ^13^C NMR spectra from 2,3-^13^C_2_ succinate-supplemented CNS perfusion fluid sample, patient D:* Chemical shifts of carbons obtained are represented above their corresponding peaks with their descriptions. In the magnifications inside the circled insets, we can observe the different ^13^C-^13^C scalar couplings measured. Peaks of lactate C1 (doublet + singlet at 185.2ppm) and succinate C1 and C4 (together as a singlet 185.1ppm) appear outside the part of the spectrum shown and have not been included, as not being relevant to the other measurements. Abbreviations: DSS = 4,4-dimethyl-4-silapentane-1-sulfonate sodium salt (the internal reference standard); Fum = fumarate; Gln = glutamine; Lac = lactate; Mal = malate; Succ = succinate. (b) *Indicative ^1^H NMR spectra from 2,3-^13^C_2_ succinate-supplemented CNS perfusion fluid sample, patient D:* Chemical shifts of protons obtained are represented above their corresponding peaks with their descriptions. In the magnifications that we see inside the circled insets, we can observe the different ^13^C-^1^H and ^1^H-^1^H scalar couplings measured. (c) *NMR labelling patterns following administration of 2,3-^13^C_2_ succinate via the TCA cycle and alternative pathways.* Blue-filled circles indicate ^13^C atoms. Red rectangular outlines indicate ^13^C-labelled metabolites detected by ^13^C NMR in cerebral microdialysates. (d) Labelled metabolite enrichment obtained by ^13^C NMR in five samples from four patients with TBI administered 2,3-^13^C_2_ succinate. Values above bars represent the respective fractional ^13^C enrichment (%) of labelled metabolites: 3-^13^C glutamine, 2,3-^13^C_2_ glutamine and 2,3-^13^C_2_ lactate. 3-^13^C glutamine is presented after background-subtraction of natural abundance endogenous ^13^C (1.1%). The double-labelled molecules have not been background-subtracted as the because the probability of two natural endogenous ^13^C atoms being next to each other is 1.1% x 1.1% = 0.01%. C1 and C2 correspond to the two distinct 2,3-^3^C_2_ disodium succinate perfusions received by patient C. Not illustrated in the bar chart are 2,3-^13^C_2_ succinate (100% enriched), its first metabolite 2,3- ^13^C_2_ fumarate (100% enriched) and its second metabolite 2,3-^13^C_2_ malate (100% enriched). ME: malic enzyme; PC: pyruvate carboxylase; PDH: pyruvate dehydrogenase; PEPCK: phosphoenolpyruvate carboxykinase; PK: pyruvate kinase. (Adapted from Jalloh et al., 2017 under a Creative Commons License (CC BY),^31^ published by Sage Publications, copyright the Authors.)

Nuclear magnetic resonance (NMR) analysis was performed on 24-hour pooled cerebral microdialysis samples from five administrations of 2,3-^13^C_2_ succinate in four patients (B, C1, C2, D and E). After analysis for LPR, there was insufficient remaining sample, in Patient A, to carry out NMR analysis. All ^13^C NMR spectra showed a pattern similar to the illustrative example in Figure 4(a) (Patient D). In all five samples, 2,3-^13^C_2_ succinate and 2,3-^13^C_2_ fumarate appeared as singlets, because in both succinate and fumarate molecules, C2 and C3 are magnetically equivalent (as the molecules are symmetrical) and thus C2 and C3 appear together in the spectra, at 138 ppm for fumarate C2 and C3, and at 36.9 ppm for succinate C2 and C3. All five ^13^C NMR spectra displayed strong signals for 2,3-^13^C_2_ malate, with a doublet for the C3 (centred at 45.2 ppm, J_C3-C2_ = 37.6 Hz) and a doublet for the C2 (centred at 73.1 ppm, J_C3-C2_ = 37.6 Hz), but no singlets. Malate is an asymmetric molecule, so C2 and C3 are magnetically non-equivalent. The presence of doublets and absence of singlets indicates that all of the observable malate was double-labelled, and derived from the perfused 2,3-^13^C_2_ succinate. For glutamine (an asymmetric molecule), doublets for C3 and C2 were clearly visible in four of the five spectra indicating the presence of 2,3-^13^C_2_ glutamine, and singlets corresponding to C3 and C2 of glutamine were seen, within each of the doublets. A singlet (in the case of an asymmetric molecule) means that the ^13^C atom does not have an adjoining ^13^C atom within the same molecule. One spectrum, from Patient C’s second perfusion period (260 h post-injury), did not show any measurable signals for glutamine C2 or C3 above the baseline noise, although this patient showed both 3-^13^C glutamine and 2,3-^13^C_2_ glutamine in his first perfusion period (40.5 h post-injury). In all five spectra, no ^13^C signals above baseline noise were seen for C4 of glutamine, which would have occurred at 33.7 ppm. Figure 4(c) is a schematic of metabolism of 2,3-^13^C_2_ succinate via the TCA cycle and spin-out pathways.^31^ The double-labelling pattern in glutamine is observed because it is synthesized from glutamate derived from alpha-ketoglutarate, a TCA cycle intermediate. Fumarate is a TCA cycle intermediate derived from succinate and malate is a TCA cycle intermediate derived from fumarate. Lactate was also observed in all five samples’ spectra, seen as doublets for C3 and C2, proving the presence of 2,3-^13^C_2_ lactate by spin-out (cataplerosis) from the TCA cycle. Singlets corresponding to C3 and C2 of lactate were also observed, within each of the doublets.

#### ^1^H NMR spectroscopy allows calculation of fractional enrichment of metabolites

We measured the endogenous concentration of these metabolites using ^1^H NMR spectra. The water-suppressed ^1^H NMR spectra obtained for the five samples showed a peak pattern similar to that for Patient D in Figure 4(b). In all five ^1^H spectra, fumarate was a wide doublet (centred on 6.5 ppm, J_13C-1H_ = 157.7 Hz). The lack of a ^1^H signal in the centre of this doublet indicates that endogenous ^12^C fumarate was undetectable, and therefore the fumarate signals arise entirely from the administered 2,3-^13^C_2_ succinate perfusion. The ^1^H spectra showed succinate as a very large doublet (centred on 2.4 ppm, J_13C-1H_ = 123.2 Hz) much greater than any central peak inside the doublet, suggesting that any endogenous ^12^C succinate was negligible compared to the exogenous 2,3-^13^C_2_ succinate administered. Both 2,3-^13^C_2_ fumarate and 2,3-^13^C_2_ succinate are thus regarded as 100% ^13^C enriched. ^1^H spectra showed no clear signals for endogenous ^12^C malate, consistent with findings for malate in ^13^C NMR spectra showing that observable malate was all derived from exogenous 2,3-^13^C_2_ succinate, and therefore that 2,3-^13^C_2_ malate was effectively 100% ^13^C enriched. Endogenous ^12^C glutamine and lactate ^1^H NMR signals were observed in all five samples. The ^1^H multiplet at 2.1 ppm (protons attached to C3 of glutamine) was used to quantify [^12^C] for glutamine, and the doublet at 1.31 ppm was used to quantify [^12^C] for lactate (protons attached to C3 of lactate), for subsequent ^13^C fractional enrichment calculations.

Results for ^13^C fractional enrichment (%) are shown in Figure 4(d) for 2,3-^13^C_2_ glutamine 2,3-^13^C_2_ lactate, and 3-^13^C glutamine. In addition, the ^13^C fractional enrichments of 2,3-^13^C_2_ succinate, 2,3-^13^C_2_ fumarate and 2,3-^13^C_2_ malate were effectively 100% in all five samples. The medians for enrichment were 6.6% for 2,3-^13^C_2_ glutamine and 0.9% for 2,3-^13^C_2_ lactate, using the C3 doublet for the ^13^C measurements in each case and the individual values shown in Figure 4(d). ^13^C enrichment (median 1.6%, range 0–2.6%) was observed for C3 glutamine singlet (median 1.6%, range 0–2.6%) and C2 glutamine singlet (median 0.6%, range 0–1.3%) revealing metabolic recycling of 2,3-^13^C_2_ succinate’s metabolites. However, ^13^C enrichments were negligible in lactate C3 singlet and in lactate C2 singlet, in all five samples.

## Discussion

### Multimodality monitoring can identify distinct neurometabolic states

We have demonstrated that utilising a tiered clinical protocol based on multimodality monitoring parameters, several neurometabolic states can be identified following TBI. There have been retrospective studies combining different microdialysis metrics in order to establish specific pathological states.^38^ Nordström and colleagues defined “ischaemia” as LPR > 30 and pyruvate <70 µmol/L, while “mitochondrial dysfunction” was defined as LPR > 30 at pyruvate ≥70 µmol/L in severe TBI patients.^13^ Similarly, Stein and colleagues defined a state entitled “metabolic crisis” when cerebral glucose levels were <0.8 mmol/L and LPR > 25.^39^ We believe that these classification algorithms are limited by not considering other common physiological TBI derangements than MD that have been shown to result in deranged LPR in prospective studies, such as high ICP, and low CPP or PbtO_2_.^38,40,41^ However, these studies did not follow a structured, tiered protocol as in the current study specifically targeting LPR. Overall, while different pathological states incorporating microdialysate markers have been described, this is the first study to fully incorporate other multimodal metrics using a tiered therapeutic protocol targeting LPR establishing a state that we can reliably attribute to mitochondrial dysfunction. We have also been able to identify the frequency of alternative derangements in metabolism may be amenable to specific intensive care interventions.

The commonest pattern seen was “normal” LPR (<25), however, in those with LPR > 25, MD and neuroglycopaenia were the most common NMS. The relative paucity of intracranial hypertension, delivery failure and tissue hypoxia in relation to mitochondrial dysfunction may reflect the success of modern neurointensive care in identifying and treating these physiological derangements. An NMS was only categorised when this abnormality could be identified in at least 2 contiguous hourly assessments. Given that ICP and brain tissue oxygen were actively targeted in a goal-directed fashion, the low incidence of these metabolic states reflects the rapid correction of any abnormalities within 2 hours. While neuroglycopenia resulting in higher LPR has been acknowledged in patients with low serum glucose,^42^ this treatment modality is more controversial and to target a threshold of up to 10.0 mmol/L serum glucose is more complicated as this metric is not monitored as frequently.

A substantial majority of patients (73%) demonstrated a multimodality monitoring signature compatible with MD at some point during their neurointensive care stay. As we have classified MD as the NMS where all other monitoring parameters are within a normal range, our study protocol systematically corrected derangements in multimodal parameters and we have selected for LPR > 25 which is resistant to standard neurointensive care interventions. In this circumstance, there is a derangement of cellular redox state despite adequate oxygen and glucose delivery, suggesting a fundamental failure of oxidative phosphorylation at the mitochondrial level. This approach is stringent in the classification of MD and we accept that it is highly likely that derangements in metabolism overlap within the same patient at different times as well as regionally, and this limits the specificity of the classification into NMS. Our averaged frequency of MD using pooled individual neuromonitoring data from all time points (21%) was similar to the biochemical pattern “mitochondrial dysfunction” that occurred during 32% of cerebral microdialysis monitoring in Nordström and colleagues’ retrospective work.^13^ We similarly found that a neurometabolic state indicative of “mitochondrial dysfunction” was more common than that of delivery failure/oxygen diffusion barrier, though Nordström and colleagues did not use PbtO_2_ monitoring.

Our use of multimodality-directed treatment has additional potential advantages. In murine models of heart attack and stroke, succinate accumulation during ischaemia has been associated with an increase in reactive oxygen species on reperfusion with exacerbation of mitochondrial damage.^43^ Our protocol specifically identified patients with normal PbtO_2_ values to avoid the risk of administering succinate during ischaemic conditions that might risk the production of reactive oxygen species on restitution of oxygen delivery. However, recent evidence from both cellular and animal mitochondrial models demonstrates a complex involvement of succinate in both reactive oxygen species’ production and elimination regardless of underlying oxygen levels.^44^ The role of neuroinflammation and ingress of inflammatory cells as a mechanism of raised LPR^45^ has not been addressed in this study. Succinate itself may also potentiate neuroinflammation in certain circumstances,^46^ adding additional complexity to the potential interaction between neuroinflammation and metabolism. Irrespective of this, the effects of succinate administration on LPR within 24 hours, and the NMR labelling patterns of downstream metabolites both suggest a direct metabolic effect of administered succinate.

### Microdialysis delivery of disodium succinate can improve local neurometabolic state

Having identified MD by multimodal monitoring criteria, we delivered micro-doses of succinate using cerebral retrodialysis into the brain region being monitored.^31^ In the present study we have demonstrated that this administration led to a modest but statistically significant increase in cerebral lactate (+5.3%), pyruvate (+20%) and glucose (+17%). We interpret this as the exogenous succinate entering the TCA cycle, providing additional carbon skeletons and sparing upstream pyruvate and glucose. Furthermore, there was a statistically significant reduction in PbtO_2_ (−5%) and the LPR (−12%) between baseline and perfusion levels suggesting that the succinate entering the TCA cycle has been successfully metabolised oxidatively, leading to an improvement in cellular redox state. These findings are consistent with our other studies of retrodialysis succinate administration in TBI.^11,31^

### Neurometabolic significance of succinate administration

The absolute amount of succinate delivered by the catheter in each 24-hour administration is small and is free to diffuse away from the cerebral microdialysis catheter into the surrounding brain. This limits the magnitude of change on multimodality parameters from the administered succinate. Double ^13^C-labelling demonstrates unambiguously that substrate molecules (2,3-^13^C_2_ succinate administered as a disodium salt) diffused from the perfusate into the brain extracellular space, entered cells and were metabolised, exported into the extracellular fluid, and were recovered by the microdialysis catheter.^31^ Earlier literature indicates that cells take up succinate via solute carrier family 13 (SLC13) Na^+^-coupled di-carboxylate and tri-carboxylate transporters.^47–49^ SLC13 transporters occur widely, including in brain astrocytes and neurons where succinate uptake and metabolism were shown using radio-labelling.^50–52^ Nonspecific uptake might occur with increased plasma membrane permeability.

Exogenous 2,3-^13^C_2_ succinate produces ^13^C double labelling in metabolites with the following ^13^C fractional enrichments: 2,3-^13^C_2_ fumarate (100%), 2,3-^13^C_2_ malate (100%), 2,3-^13^C_2_ glutamine (6.6%), and 2,3-^13^C_2_ lactate (0.9%), thus all at several orders of magnitude higher than what would be expected naturally (0.01%). Notably, all detectable fumarate, the metabolite directly downstream of succinate, originated from the exogenous succinate administration indicating complex II activity from the administered succinate.

Lowering of LPR by succinate administration suggests improved redox balance, conceivably by boosting shuttles utilising mitochondrial ETC to recycle NADH to NAD^+^, placing less reliance on conversion of pyruvate to lactate in the cytosol. Extracellular LPR is an indicator of the cytosolic NADH/NAD^+^ ratio,^53,54^ which is in turn influenced by mitochondria, as summarised previously.^31^ NADH itself cannot cross the mitochondrial membrane, so the requisite hydrogens and electrons are transferred indirectly by “shuttles” (malate-aspartate and glycerol-3-phosphate).^55^ Malate-aspartate shuttle operation concurs with occurrence of 2,3-^13^C_2_ malate in microdialysates when 2,3-^13^C_2_ succinate is administered. The 2,3-^13^C_2_ lactate labelling pattern we observed is the same as in our previous 2,3-^13^C_2_ succinate microdialysis study.^31^ 2,3-^13^C_2_ lactate suggested the TCA cycle spinout of 2,3-^13^C_2_ pyruvate (from 2,3-^13^C_2_ malate by malic enzyme, or from 2,3-^13^C_2_-oxaloacetate by phosphoenolpyruvate carboxykinase and pyruvate kinase), then LDH-mediated conversion to lactate. In earlier literature, TCA cycle spin-out of lactate was reported in animal brains.^56–58^

We have previously combined in-vivo voxel-based magnetic resonance spectroscopy (MRS) and microdialysis in TBI patients, to demonstrate that a reduction in LPR correlates significantly with an increase in PCr (phosphocreatine)/ATP ratio measured by MRS (Spearman's rank correlation, r = −0.86, p = 0.024).^11^ Our findings support the interpretation the reduction in LPR is directly linked to brain energy state, and that succinate may support brain energy metabolism in select TBI patients suffering from mitochondrial dysfunction.

Other combined imaging/microdialysis studies using positron emission tomography (PET) have demonstrated that oxygen extraction fraction correlates with lactate/pyruvate ratio^59^ and that derangements in LPR can occur without classical ischaemia^60^ reinforcing the findings in this study.

### Clinical implications

Cerebral microdialysis is a focal monitor and we cannot make any assumptions about the NMS in areas remote from the volume of brain monitored. However, all the monitors that were utilised and the delivery of succinate by retrodialysis were to the same volume of brain. In this way, this experimental medicine approach has used the volume of monitored brain as a microcosm of the injured brain as a whole. The frequent identification of MD in this study suggests that mitigating its effects is a potentially fruitful therapeutic avenue in a multimodality-monitoring defined cohort. We hypothesise that systemic supplementation of succinate with a higher absolute dose has the potential to have a greater impact on cerebral metabolism by providing an alternative metabolic fuel to the injured brain, whether following TBI or other neurological insults. The present study has administered succinate focally to a small group of patients and utilised within-patient comparisons. Our approach does not allow us to explore the effects of succinate on clinical outcome. We have delivered very small amounts of succinate into a small volume of brain around the microdialysis catheter, and we would not expect this to have any clinically significant therapeutic effect on the brain as a whole.

In order to demonstrate wider applicability of succinate as a therapeutic strategy with clinical outcome measurements, much larger studies will be required. Administration of succinate prodrugs have shown efficacy in *ex vivo* human tissue in theoretical management of metformin-induced lactic acidosis, where metformin inhibits Complex I of the TCA cycle causing lactate accumulation.^61,62^ However, these prodrugs have not been used in any clinical trials to date, but could become a tentative treatment option in the future. Furthermore, systemic administration of succinate itself (rather than a prodrug) *in vivo* may also have therapeutic potential in TBI for MD. Oral succinate (2x500 mg doses per day) has been given to alleviate the symptoms of mitochondrial complex I deficiency in children.^63,64^ Also, oral succinate (6 g/day) was given to an adult patient suffering from MELAS (mitochondrial myopathy, encephalopathy, lactic acidosis and stroke-like episodes), and to another adult patient with Kearn-Sayre syndrome (succinate 6 g/day plus Coenzyme Q10 300 mg/day), which in both patients resulted in clinical improvement and no adverse events.^65,66^ In any future studies of systemic delivery of succinate in TBI, as with the present approach of focal delivery, it is vital to ensure that there is adequate oxygen supply, to avoid the risk ischaemia-reperfusion injury, as we have discussed previously.^31,43^

## Conclusions

Together, the findings from the present study demonstrate how a clinical protocol incorporating multimodality monitoring data can be utilised to identify specific metabolic abnormalities and target appropriate therapies that improve metabolic derangements following human TBI. Exogenously administered succinate was able to enter the TCA cycle, was metabolised oxidatively, and improved cellular energy metabolism as measured by cerebral LPR. Supplementation with succinate or other mitochondrially-active species merits further investigation for TBI therapy.

## Supplemental Material

sj-pdf-1-jcb-10.1177_0271678X211042112 - Supplemental material for Focally administered succinate improves cerebral metabolism in traumatic brain injury patients with mitochondrial dysfunction Click here for additional data file. Supplemental material, sj-pdf-1-jcb-10.1177_0271678X211042112 for Focally administered succinate improves cerebral metabolism in traumatic brain injury patients with mitochondrial dysfunction by Abdelhakim Khellaf, Nuria Marco Garcia, Tamara Tajsic, Aftab Alam, Matthew G Stovell, Monica J Killen, Duncan J Howe, Mathew R Guilfoyle, Ibrahim Jalloh, Ivan Timofeev, Michael P Murphy, T Adrian Carpenter, David K Menon, Ari Ercole, Peter J Hutchinson, Keri LH Carpenter, Eric P Thelin and Adel Helmy in Journal of Cerebral Blood Flow & Metabolism
