# Supplementary material for: Focally administered succinate improves cerebral metabolism in traumatic brain injury patients with mitochondrial dysfunction
Source: J Cereb Blood Flow Metab. 2021 Sep 8;42(1):39–55. doi: 10.1177/0271678X211042112 (PMC8721534; doi:10.1177/0271678X211042112)
Supplement: sj-pdf-1-jcb-10.1177_0271678X211042112 - Supplemental material for Focally administered succinate improves cerebral metabolism in traumatic brain injury patients with mitochondrial dysfunction [file sj-pdf-1-jcb-10.1177_0271678X211042112.pdf]

Supplementary Materials

Focally Administered Succinate Improves Cerebral Metabolism in Traumatic Brain Injury Patients with Mitochondrial Dysfunction

Supplementary Table 1. Patient demographics of the succinate cohort.

TBI patient ID	Age (years)	Sex	Mechanism of injury	GCS at scene (/15)	2,3-$^{13}C_2$ Disodium succinate perfusion period start time (hours from injury)	Intraparenchymal catheter location	Admission CT head scores			Description of injury on admission CT head
							Marshall (I-VI)	Rotterdam (1-6)	Stockholm	
A	20	M	MVA	8	52.5	R frontal	III	5	2	SAH, pneumocephalus, skull (including BOS) fractures, facial fractures
B	42	M	Fall	8	177	R frontal	IV	3	2.6	SAH, contusions, skull fractures
C	24	M	MVA	6	1st dose: 40.5<br>2nd dose: 260	R frontal	III	3	2	R ASDH, SAH, contusions
D	21	M	Fall	3	35.4	R frontal	III	2	1	R ASDH, contusions
E	56	M	Fall	14	203	R frontal	II	2	1.5	L ASDH, SAH, contusions, BOS fracture

Table describing the demographics from the patients receiving 2,3-$^{13}C_2$ disodium succinate by cerebral retrodialysis. Succinate-supplemented perfusion fluid was administered for 24 hours in each case. Computerised tomography (CT) scoring systems were individually noted as per previously described methodology [65-67]. Abbreviations: ASDH = acute subdural haematoma; BOS = base of skull; GCS = Glasgow Coma Scale; ID = identification; L = left; M = male; MVA = motor vehicle accident; R = right; SAH = subarachnoid haemorrhage; TBI = traumatic brain injury.
